# The NQRN Registry Maturational Framework: Evaluating the Capability and Use of Clinical Registries

**DOI:** 10.5334/egems.278

**Published:** 2019-07-17

**Authors:** Seth Blumenthal

**Affiliations:** 1 American Medical Association, US

**Keywords:** Registries, Clinical Registries, Patient Registries, Clinical Data Registries, Qualified Clinical Data Registries, QCDRs, Electronic Health Records, EHRs, Quality Improvement, Health Information Exchange, Health Services Research, Data Collection, Maturational frameworks, maturity models, classification

## Abstract

Clinical registries are increasingly used as national performance measurement platforms. In 2018, nearly 70 percent of the more than 50 specialty society registries in the United States were used by the Centers for Medicare & Medicaid Services (CMS) to measure the quality of clinical care. Private payers and evaluating organizations also use or desire to use registry information to inform quality improvement programs and value-based payment models.

The requirements for an entity to become a CMS Qualified Clinical Data Registry (QCDR) constitute a minimum set of standards for the purpose of reporting to the CMS Quality Payment Program. Models and frameworks exist that can help classify registries by purpose and use, and maturity models are available for evaluating health IT systems generally. However, there is currently no framework that describes the capability that should be expected from a registry at different phases of its development and maturity.

In response, the National Quality Registry Network (NQRN) has developed a registry maturational framework. The framework models early, intermediate and mature development phases, the capabilities anticipated during these phases and 17 domains across which registry programs support those capabilities. The framework was developed and refined by NQRN registry stewards, users and other stakeholders between 2013–2018. It is intended to be used as a developmental guide or for registry evaluation. The successful use of registry information to execute value-based payment models is a critical need in U.S. health care. The NQRN framework can help ensure that our national system of registries is rising to the occasion.

## Introduction

A clinical registry is an organized system for the collection, analysis and reporting of clinical data about patient care for the improvement of health outcomes [1]. With the increased use of registries for research, performance evaluation, quality improvement and payment, there is corresponding interest in new registry development and increasing the functionality of established registries. The National Quality Registry Network (NQRN) is a community of organizations interested in clinical registries. NQRN is a program of the PCPI Foundation, a national convener of medical specialty and health care professional societies and associations [2]. To accelerate these trends and guide registry development and use, NQRN has developed a maturational framework for clinical data registries.

**Figure 1:**
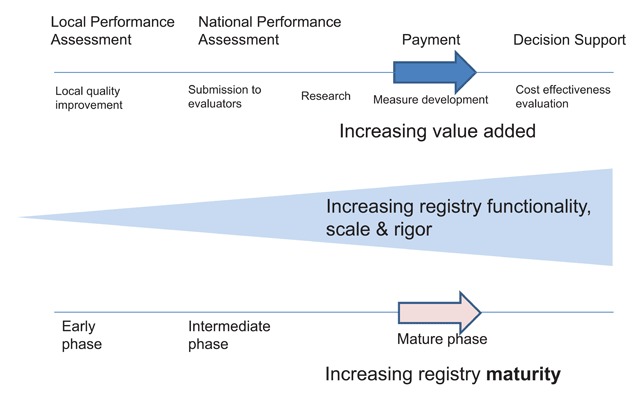
How Registries Add Value to Health Care.

Clinical registries have made many contributions to the health care knowledge system [3,4,5,6,7,8,9]. As registry data collection, analysis and reporting capabilities have improved, their use in improving health system effectiveness has expanded (Figure 1). Today’s patchwork system of registries is moving toward a coordinated and transparent system, with improved interoperability envisioned between registries and other health information systems to support better efficiency and outcome evaluations. Additionally, registries have a need to be able to demonstrate the high quality of their data through transparency of performance measures and quality control techniques.

Although the United States leads the world in national health care expenditures, robust data to support quality improvement, accountability and consumer engagement are lacking [10]. As clinically rich data have become more widely available, there is interest in leveraging these data to improve the overall quality and efficiency of the U.S. health care system [11].

A clinical registry is utilized for many purposes including assessing performance; determining clinical, cost or comparative effectiveness; and measuring or improving the quality, safety or efficiency of care. Specific applications include surveillance, clinical practice guideline development, appropriateness determination, performance evaluation and improvement monitoring, research and public reporting.

All data sources – administrative claims, clinical information systems, electronic health records (EHRs) and registries – have advantages and limitations. Although originally developed for billing and other administrative functions, claims data have subsequently been utilized for a wide variety of purposes including performance evaluation due to their availability, low cost, standardized coding systems such as the International Classification of Diseases (ICD) and Current Procedural Terminology (CPT), and the large volume of available data they represent. However, administrative data often lack clinical granularity and specificity, may be difficult to aggregate and analyze across payers, and have been viewed skeptically by the provider community when used for performance evaluation.

Clinical information systems and EHRs also have advantages and limitations compared with registries. Their data models have been designed to support direct patient care and operations, with a secondary focus on performance evaluation. Roski et al. estimate that, “…less than 15 percent of health data in EHRs might be entered in structured data fields that allow those data to be analyzed using traditional retrieval and analysis methods [12].” A significant proportion of the data captured in these systems lack standardized data definitions, thus limiting their usefulness for the purposes articulated above.

Registries are designed by clinical experts and contain data that are clinically relevant, structured, precisely-specified, understandable, and acceptable to health care providers. Registries typically include data from real-world populations and multiple payers, distinguishing them from many administrative data sources. For research, registry data may offer an advantage over traditional randomized clinical trials as rigid study population inclusion and exclusion criteria in the latter may limit the generalizability of their results. Finally, registry data are collected using processes that ensure data validity and good inter-rater reliability, with data quality audits ensuring ongoing accuracy.

Registries also have limitations. Currently, most registries focus on short-term outcomes making it difficult to assess care over time. They commonly lack information on readmissions, re-interventions, medication adherence, patient functional status and cumulative costs. This information is becoming increasingly important as responsibility for outcomes and costs extends well beyond a single episode of care.

Given these considerations, one approach may be to combine the positive attributes of each data source, while mitigating their respective weaknesses [13]. To accomplish this, commonly used data elements will need to be standardized to facilitate data linkages [14]. For example, using the registry as the foundation, linkages could be established to EHRs for demographics, to EHRs or clinical information systems for laboratory and diagnostic results, and to administrative claims and other data sources for repeat hospitalizations, procedures, professional services, cost information and long-term survival [15,16].

## The Need for a Registry Maturational Framework

There are over 60 national clinical registry programs in the United States, many of which are recognized for the quality of their data and their contributions to improved patient care [17]. While they are typically developed by professional organizations, the value of registries is increasingly recognized by payers. For example, the Medicare Access and Reauthorization Act of 2015 (MACRA) required the Centers for Medicare & Medicaid Services (CMS) to expand the options for registry data submission in the Quality Payment Program (QPP), which consolidated earlier programs such as the Physician Quality Reporting System. Since 2014, CMS has certified over 50 national clinical registries as Qualified Clinical Data Registries (QCDRs). Certification as a QCDR ensures that a registry meets stringent, national requirements. Registries are also being utilized by the U.S. Food and Drug Administration for post-marketing surveillance of medical devices [18].

## Prior Work on Clinical Registry Evaluation

There is a growing body of work and increasing interest in registry description, classification and evaluation. In 2012 the NQRN conducted a registry landscape assessment which identified multiple unmet needs of registries, as well as variation in governance and operational processes. The survey was conducted a second time in 2015 [19]. In addition to these efforts, others have offered models and frameworks to help define and classify registries. In 2008 Drolet et al published a classification system for registries that identified five characteristics of a registry, using the term in its broadest sense: mergeable, standardized longitudinal data from multiple users, including outcomes, collected using a rules-based approach [20]. Klaiman et al examined registry use in 2013 and found that “effective registries were successful in 1 or more of 6 key areas: data standardization, transparency, accuracy/completeness of data, participation by providers, financial sustainability, and/or providing feedback to providers [21].” The Australian Commission on Safety and Quality in Health Care has published a framework for quality registries that describes desired registry attributes across domains including data collection, risk adjustment, data security, data quality, governance, ethics & privacy, reporting and funding [22]. A 2016 report by Crowley et al offered a Public Health Information Technology Maturity Index. Although not specific to registries, the index offered a maturity dimension in addition to domains including scale and scope, quality, human capital, policy, resources and infrastructure [23].

At the time of publication there was no national standard for evaluating the maturity or performance of registries in general. The QCDR requirements could be considered a baseline at least for reporting performance measure results to evaluating organizations for the purpose of carrying out value-based payment programs. Health Level Seven International (HL7), a standards developing organization, publishes domain analysis models on subjects of interest to the health care data standards and informatics communities. A domain analysis model contains an abstract representation of a specific subject area, oriented to the needs of health IT implementers and users [24]. As part of its Common Clinical Registry Framework project, in 2017 HL7 published a model that “…describes the function, organization, structure and major workflows of a general clinical registry” [25]. As a set these models and frameworks provide useful tools for describing and classifying registries, however none include a maturity model developed specifically for evaluating registry capability through multiple phases of maturity.

In response to the variations in registry development identified in its registry landscape surveys and given the lack of a targeted maturity model, the NQRN developed a clinical registry maturational framework (Framework) to guide the development of registries over time.

## Methods

Development of the Framework began with the identification of important registry domains such as registry use, scope, data quality and performance measurement. Early, intermediate and mature developmental stages were then described for each domain, and the resulting intersecting cells were populated to demonstrate how a registry might evolve over time. The Framework was further refined with broader stakeholder input from NQRN participants in November 2013, as well as further updates by NQRN staff in 2016 and 2018.

## Description and Use

The Framework describes the domains in which a registry might mature over time. The relative importance of each domain is based on the use(s) of the registry. The Framework is a roadmap for achieving the highest level of value from a registry and a guide to identifying the underlying capabilities and infrastructure that will be necessary to achieve that value.

The Framework has been developed according to a set of general principles: (1) it is intended to be a guide to registry development, not a standard; (2) content is organized by topical domain and phase of maturity; (3) content is generally cumulative as maturity increases within a given domain; (4) at any given time, a registry may be at different stages of maturity across the domains; and (5) the desired final stage of maturity in a domain may differ depending on the mission of the registry.

Within the Framework, registry activities are generally cumulative as maturity increases within each domain. However, not all registries will mature along the same trajectory, with differences driven by the needs of its participants and end users. Finally, the Framework reflects the current state of registry development which is expected to evolve over time as medical science, payment models and information technologies advance.

## The Topical Layer: Registry Domains

Registries can be described by applying a model comprised of different domains – starting with registry use, followed by additional domains describing underlying capabilities at each phase of maturity (Table 1).

**Table 1:** Maturational Framework Domains.

Domain	Definition

Registry use	Activities the registry is used for or developed to support
Stakeholder relationships	Relationships that registry stewards establish in order to develop and maintain a registry
Legal, ethical & oversight	Legal and ethical aspects related to a registry’s governance and operations
Participation size & scope	The representativeness of a registry’s patient population
Data type	Clinical concepts and metadata captured in a registry
Data capture, storage & transmission	The level of automation and standardization for data capture and transmission to and from a registry
Data model & quality	Capabilities and methods that influence the ability of a registry to capture high-quality data despite variances in sample size, validity and reliability
Measure development & use	The types of performance measures used by a registry, along with its contribution toward measure development efforts
Measure type	The kinds of clinical and other concepts measured by a registry
Data collection basis	The time domain scope of registry data collection
Reporting frequency	The frequency with which the registry provides reports to its participants
Public reporting	Registry reporting intended for public audiences
Transparency	Details of registry design, activities and operation that are made available to the public
Quality improvement	Registry reports, other performance-related feedback to participants including programmatic activities intended to improve the performance of health care operations
Research	Capabilities and infrastructure to support the use of registry information for clinical research
Human capital needs	Recommended skills and capabilities to have either in staff or outsourced resources
Registry networks	The degree to which the registry participates in professional and data-sharing networks

## The Structure for Development: Phases of Maturity

**Figure 2:**
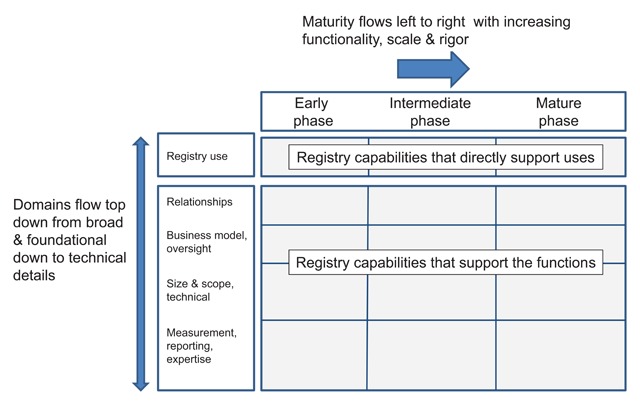
A Maturational Framework for Registries.

For a registry to expand its usefulness and overall maturity, increased supporting capabilities are needed. The Framework takes a developmental staged approach to describe what to expect of a registry at different phases of development, along with the capabilities needed. In the early phase of development, a registry typically begins to provide value to participants who use registry data for local quality improvement or performance assessment and reporting. A registry in the intermediate phase has achieved a critical mass of participation to support performance measurement and other activities with increased scope and rigor. Mature registries provide data to support health system decision making at a national or international level as well as for research and health care policy. Registries may achieve different maturity phases across the various domains at any given period of time, but some congruence is anticipated due to the cumulative advancement of underlying capabilities needed to support particular uses in the next higher phase. Examined together, the topical domains and the development structure allow a registry to be assessed holistically. (Figure 2).

A table containing the complete Framework matrix is in Appendix A.

## Suggested Uses

The Framework is intended to guide registry development; it is not a set of standards or requirements. It is hoped that its guidance will facilitate the development of registries in a logical fashion and support the adoption of leading practices and national standards at an accelerated pace.

The Framework has many potential uses. It can guide registry stewards along a consistent path to maturity with a shared understanding of what the different stages of maturation entail. It can be used as a general guide to understand what might be needed for a registry to qualify for use by a private or public payer such as CMS through its QPP. The Framework can help users of registry data e.g., health systems, researchers, evaluators and payers assess a registry’s capabilities. It can also be used by registry stewards to communicate with consumers about how information was collected, analyzed and reported.

The Framework may be a useful assessment tool for registry stewards, those planning a new registry, and users of registry information. The Framework can be used to define the goals of the registry and the developmental pathways to achieve these goals. It is a valuable resource to identify a registry’s strengths, areas where additional focus and resources may be needed, and actions required to expand a registry’s purposes.

With its strong emphasis on descriptiveness combined with flexibility that allows each registry to fulfill its stated purposes, the Framework can provide valuable insight to accelerate the pace and quality of registry development. An existing registry can evaluate its maturity level and gain insight that can help prioritize further development efforts. A new registry can better understand the capabilities that will support its mission. Users of registry data for research, performance improvement and payment can better understand the level of rigor and scope they can expect from the data. There are many other potential uses (Table 2).

**Table 2:** Examples of Potential Uses of the Framework.

Situation	Goal	How the Framework helps

A growing registry is using manual entry data submission that is costly for its participants	Change over to automated data submission from participants’ EHRs	Helps the registry understand the underlying functionality that should be developed in order to support improved interoperability via automated data extraction from electronic sources
A registry seeks to measure clinician performance on a national level	Develop national standard measures	Helps the registry understand the expertise and capabilities that support measure development
A health plan wants to use registry data for quality evaluation and wants to understand what it can expect from a registry	Evaluate registries for program participation	Provides a context for evaluating the quality, scope and rigor of registry data. Payers can use this information to develop participation requirements.
An organization is being asked to submit data that support the execution of value-based payment models	Understand the capabilities needed in a registry that participates in payment programs	Outlines these capabilities and provides a path to achieving them

## Limitations

Although the Framework is comprehensive and offers guidance to potentially any registry developer, it has limitations. The information it provides, while important and valuable, is necessary but ultimately not sufficient for a sustainable and credible registry to emerge and evolve. The Framework as currently bounded does not address several relevant dimensions such as a) governance and ownership of registries and registry data, b) registry business models and access/use requirements, c) a range of technical issues such as execution of data use agreements to whether data is distributed, consolidated in one place, or some hybrid, and d) approaches to privacy protection. As specialty societies and other registry stewards continue their work in developing and refining sustainable business models, the registry community through the NQRN and other stakeholders has an opportunity to continue the development of this Framework to address these limitations.

## Conclusion and Next Steps

It is envisioned that the uses of the Framework will expand in the future. For example, it could serve as an assessment tool for those considering participation in a registry i.e., health systems, practitioners, or health plans, consumers and others interested in using registry data for improvement and related purposes.

Supported by the NQRN and the Framework, as well as additional NQRN resources, registries are expected to continue to mature as an important data source to drive improvement in health care delivery [26]. With strategic investments in data standardization, linkages among registries and other data sources, and alignment of financial incentives, the U.S. health care system will evolve from being “data poor” to “knowledge rich.”

## Appendix A: The Framework

**Table:**

Domain	Early	Intermediate	Mature

Registry use	Quality improvement Performance assessment Performance feedback to participants	Performance measurement using nationally-accepted measures Participation in payment programs Benchmarking Guideline development Shared decision making Research	Measure development Clinical decision support Certification, licensing & credentialing Public reporting Hazard reporting Population health

Stakeholder relationships	Provider organizations	Payers Regulators Patients & consumers	Licensing & credentialing organizations Media

Legal, ethical & oversight	Federal & state legal requirements Data use & business associate agreements with participants Data security & privacy policies and procedures Legal, ethical implications of funding sources	Legal requirements for human subject research Collect and retain authorizations required by payers & evaluators	

Participation size & scope	Regional, state or national Participant & patient recruitment plan Sampling methodology 1–15% eligible participants 1–25% eligible patients	16–40% eligible participants 26–75% eligible patients	International >75% eligible participants >90% eligible patients

Data type [27,28]	Patient & practitioner demographics Allergies History Comorbidities Utilization Treatments Medications Vitals Procedures Provenance	Assessment & plan of treatment Clinical markers Laboratory, imaging Device Reported by patients Outcomes	Cost Sourced from patient-facing devices Employment/occupational data Payment sources Reference datasets

Data capture, storage & transmission	Mix of manually and automatically captured data Use of recognized standards for data security and privacy Use of recognized standards for syntactic/technical interoperability	Majority of data captured automatically Use of application programming interfaces (APIs) Use of standards for patient health outcomes Use of recognized standards for semantic interoperability of common data elements	Most or all data captured automatically Connectivity to multiple data sources Use of recognized standards for semantic interoperability of most or all data elements Participate in data standards development On-demand access to or synonymous with source data

Data model & quality	Parsimonious data model; collect just what is needed Formal data quality plan Data elements structured as appropriate Data capture tools & processes contribute to data quality, validity & reliability Sampling methodology Data cleaning on submission External comparison to source data Data quality reporting to submitters	Broader data model; collect high and medium priority data External review of data security, validity & reliability Criticality-based data quality procedures Ongoing data cleaning plan	Data model supports a “capture everything” model for single capture and multiple reuse as new uses are discovered in the future Data quality reporting to external data users Data quality is synonymous with the source data

Measure development & use	Use of internally developed and validated measures Use of nationally accepted measures	Development and testing of new measures meeting nationally accepted standards Participation in interdisciplinary and cross-setting measure development activities Development and testing of outcome measures	Stewardship of a portfolio of nationally endorsed measures Development and testing of measures harmonized across procedures and conditions Development of patient reported outcome measures Ad-hoc measure development by participants

Measure Type	Structure Process	Outcomes Appropriate use Patient reported health status or outcomes Deaths, adverse events & complications Comorbidities	Longitudinal outcomes Cost Value Patient centered

Data collection basis	Point in time	Episode of care	Longitudinal Population

Reporting frequency	Periodic with appropriate timeliness given reporting aims	At least quarterly	Real time

Public reporting	Awareness of public reporting and preparation for doing so in the next phase	Publicly report measure results on own website Reconcile cases across facilities to mitigate facility effect Safety & effectivity of procedures	Publicly report measure results through national comparison sites or health ratings organizations Measure results show meaningful differentiation Practice level Information on realistic expectations for treatments

Transparency	Governance structure & representation Data quality methodology Participation cost Data elements	Data element specifications Measure information Measure level reporting rates Risk adjustment methodology	Measure testing methods & results

Quality improvement	Local benchmarking against goals	External/comparative benchmarking Participation in or stewardship of regional QI programs	Participation in or stewardship of national QI initiatives Clinical decision support Patient education Patient engagement support

Research	Policies & procedures in development	Policies & procedures in place Contribute to national performance improvement knowledge	Participate in research data sharing networks

Human capital needs	Legal, risk management Administrative/operations IT/technical Informatics, data standards & quality Clinical Participant and population recruitment Measure development Training & support for staff involved with data capture, storage & transmission Quality reporting & payment model participation	Data integration Quality improvement methods & models Statistics & research methods Public reporting Patient education Public relations	Data analytics Patient matching Quality improvement scale & spread

Registry networks	Participation in national registry networks Submit registry information to nationally accepted inventories or lists	Contribute to national registry leading practices knowledge Contribute to projects or initiatives focused on improving interoperability	Participate in data sharing networks

## Appendix B: Subject Matter Expert Contributors

Chisara Asomugha, MD, MSPH, CMS

Anthony Asher, MD, American Board of Neurological Surgery

Carmella Bocchino, America’s Health Insurance Plans

Jeremiah R. Brown, PhD, MS, Dartmouth-Hitchcock Medical Center

Dan Campion, MBA, IQVIA

Mythreyi Chatfield, PhD, American College of Radiology

Kate Chenok, MBA, California Joint Replacement Registry

Carol Clothier, American Board of Medical Specialties

Sean Currigan, MPH, American College of Obstetricians and Gynecologists

Richard Dutton, MD, MBA, Anesthesia Quality Institute

Stephen Epstein, MD, American College of Emergency Physicians

Eden Essex, American Society for Gastrointestinal Endoscopy

Jason A. Efstathiou, MD, DPhil, Massachusetts General Hospital

Caryn Etkin, PhD, MPH, American Academy of Orthopaedic Surgeons

Nancy Foster, American Hospital Association

Sheila Heitzig, JD, MNM, American Academy of Allergy, Asthma & Immunology

Kathleen Hewitt, MSN, RN, American College of Cardiology

Stacie Jones, MPH, American College of Emergency Physicians

Danielle Jungst, American College of Chest Physicians

Norman Kahn, MD, Council of Medical Specialty Societies

Jeffrey P. Knezovich, Joint Replacement Registry

Betsy Kohler, North American Association of Central Cancer Registries

Charlotta Levay, PhD, Harvard Medical School

Flora Lum, MD, American Academy of Ophthalmology

Kristen McNiff, MPH, American Society of Clinical Oncology

Thomas Murray, American Gastroenterological Association

Frank Opelka, MD, Louisiana State University Health Sciences Center

Joyce Bruno Reitzner, MBA, MIPH, American College of Chest Physicians

Koryn Rubin, American Medical Association

Dana Gelb Safran, ScD, Blue Cross Blue Shield of Massachusetts

Jill Sage, American College of Surgeons

Lewis Sandy, MD, United Health Group

John Santa, MD, MPH, Consumer Reports

Dale Singer, MHA, Renal Physicians Association

Jean Slutsky, Agency for Healthcare Research and Quality

Katie Sommers, MPH, American Society of Plastic Surgeons

Modena Wilson, MD, American Medical Association
